# Supplementary material for: Multiscale Dynamics of Lipid Vesicles in Polymeric Microenvironment
Source: Membranes (Basel). 2022 Jun 21;12(7):640. doi: 10.3390/membranes12070640 (PMC9318666; doi:10.3390/membranes12070640)
Supplement: Supplementary file 1 [file membranes-12-00640-s001.zip › supplementary 15-06-new.pdf]

Multiscale Dynamics of Lipid Vesicles in Polymeric Microenvironment

Selcan Karaz[1], Mertcan Han[3], Gizem Akay[4], Asim Onal[5], Sedat Nizamoglu[3,4,5], Seda Kizilel[1,5,*], Erkan Senses[1,2*]

[1]*Department of Chemical and Biological Engineering, Koc University, Sariyer, Istanbul 34450, Turkey*

[2]*Koç University Surface Science and Technology Center (KUYTAM), Rumelifeneri yolu, 34450, Sariyer, Istanbul, Turkey*

[3]*Department of Electrical and Electronics Engineering, Koc University, Istanbul 34450, Turkey*

[4]*Department of Materials Science and Engineering, Koc University, Sariyer, Istanbul 34450, Turkey*

[5]*Graduate School of Biomedical Science and Engineering, Koc University, Istanbul 34450, Turkey*

Corresponding author: *esenses@ku.edu.tr; skizilel@ku.edu.tr*

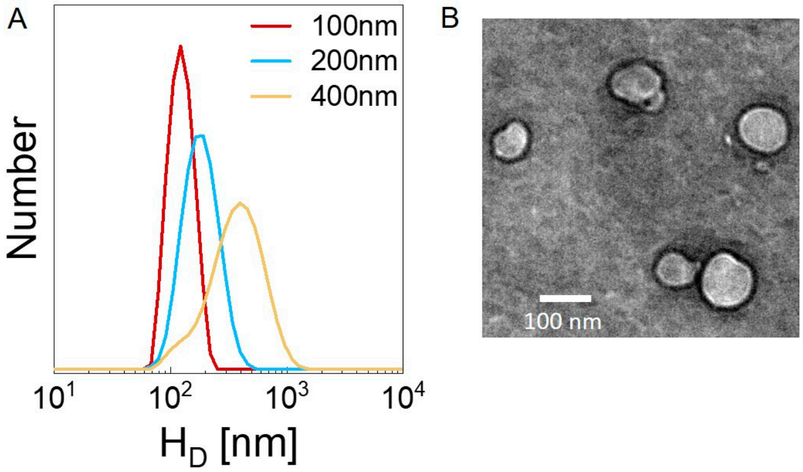

Figure S1. A. Hydrodynamic diameters of 400 nm, 200 nm, and 100 nm liposomes obtained by DLS B. Cryo-TEM images of 100 nm DMPC/DMPG liposomes.

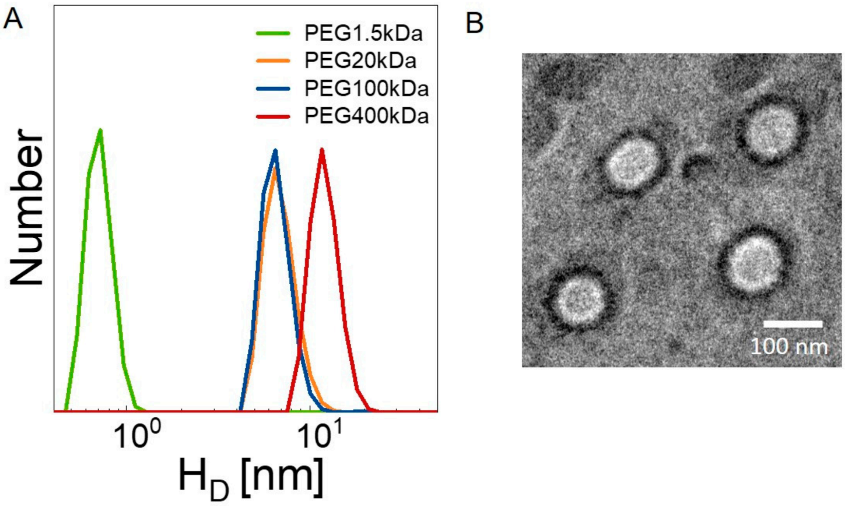

Figure S2. A. Hydrodynamic radius of PEGs with various molecular weights obtained by DLS. B. Cryo-TEM images of liposomes in 2% PEG 10kDa solution.

Table S1. Hydrodynamic diameter and PdI values of 400 nm, 200 nm, and 100 nm liposomes obtained by DLS

	Hydrodynamic Diameter(nm)	PDI
400 nm	267.2 ± 1.09	0.219 ± 0.016
200 nm	178.4 ± 9.64	0.115 ± 0.018
100 nm	119.9 ± 2.54	0.075 ± 0.010

Radius of Gyration of polymers is calculated according to the following equation;

$$R_g = \frac{< h^2 >_o}{6}$$

Where $< h^2 >_o$ (is the actual mean-square end-to-end distance of the polymer chain. $\frac{< h^2 >_o}{M}$ value is taken as 0.805 for PEG, and M is the molecular weight of the polymer. [1]

Table S2. Calculated Radius of Gyration of PEGs with various molecular weights

	Radius of Gyration (nm)	Hydrodynamic Radius(nm)
PEG 1.5 kDa	1.41	0.78±0.10
PEG 20 kDa	5.18	6.50±1.70
PEG 100 kDa	11.5	10.10±2.13
PEG 400 kDa	23.16	18.17±3.28

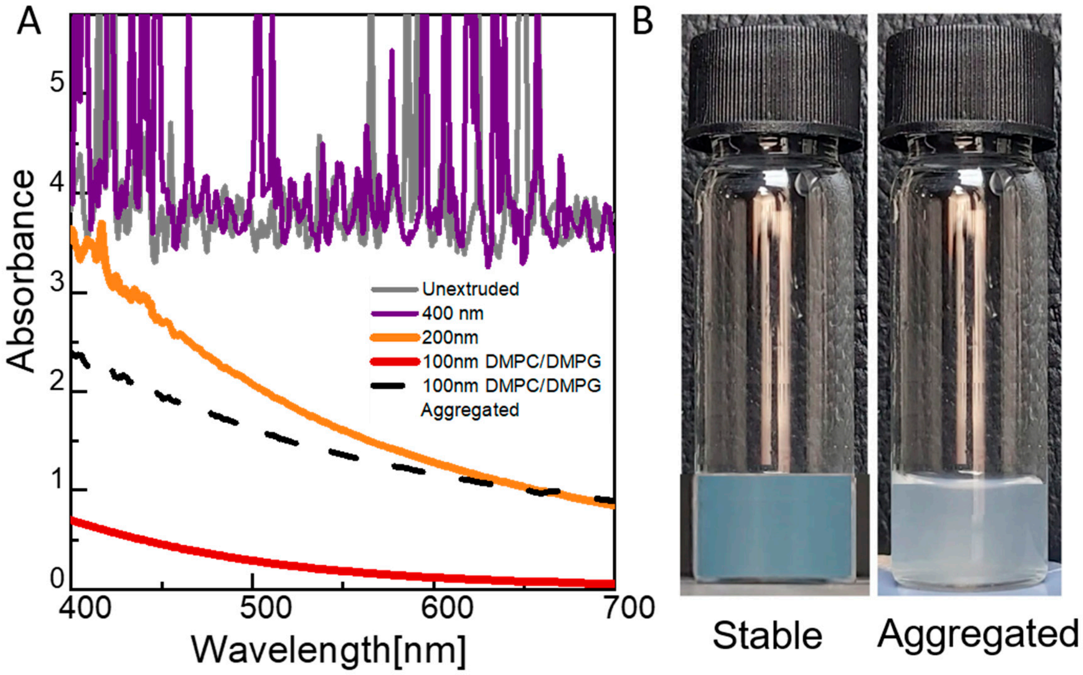

Figure S3. A. UV-vis spectra of unextruded, 400 nm, 200 nm, 100 nm liposomes, and 100 nm liposomes in PEG100kDa after 1 week preparation B. Photographs of stable and unstable (1 week after preparation) 100 nm liposomes.

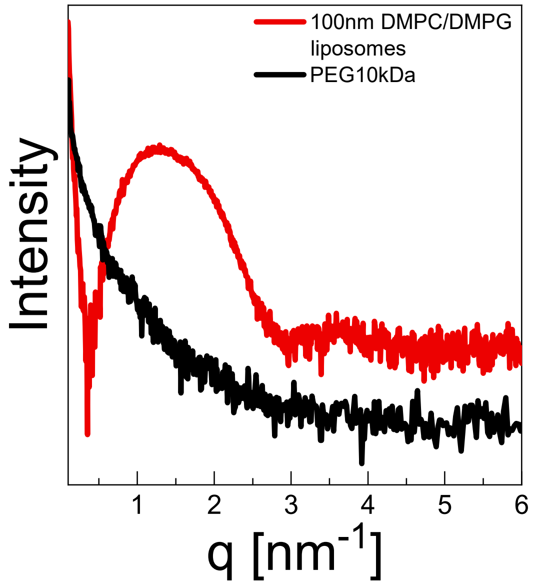

Figure S4. SAXS data of 20 mg/mL 100 nm DMPC/DMPG liposomes and 20 mg/mL PEG10 kDa

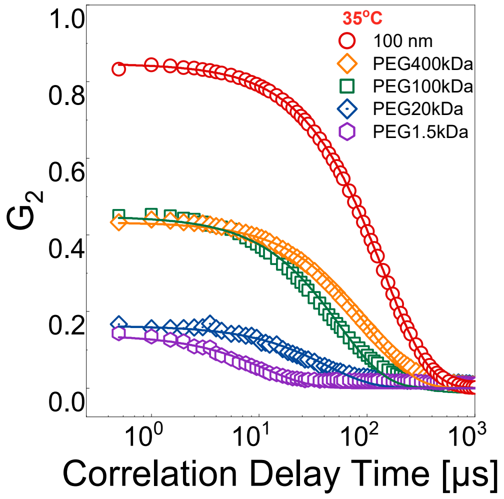

Figure S5. Autocorrelation functions of neat 100 nm DMPC/DMPG liposomes and neat PEGs with various molecular weights at 35°C.

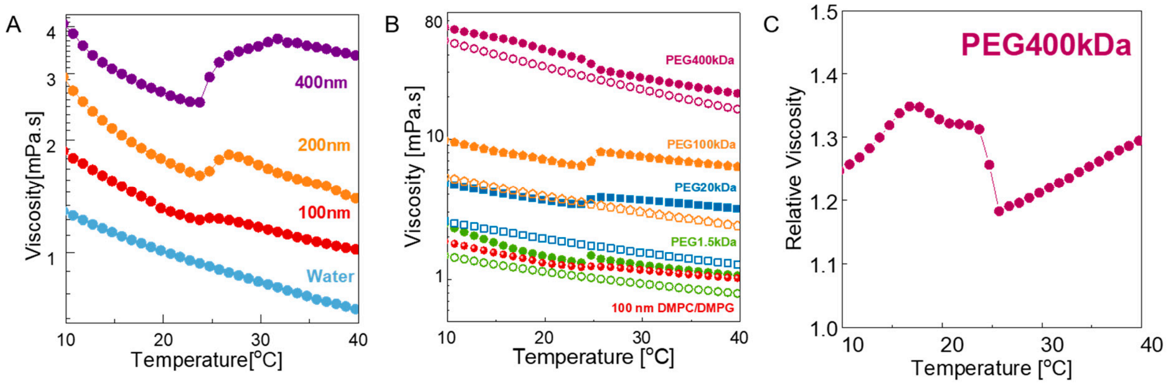

Figure S6. Viscosity of A. 2% 400 nm, 200 nm, and 100 nm liposome solutions and water B. composite solutions containing 1.7% 100 nm liposome and 1.7% PEG in water. (Empty symbols represent pure 1.7% PEG solutions) C. Relative viscosity of composite solutions containing 1.7% 100 nm liposome and 1.7% PEG 400 kDa.

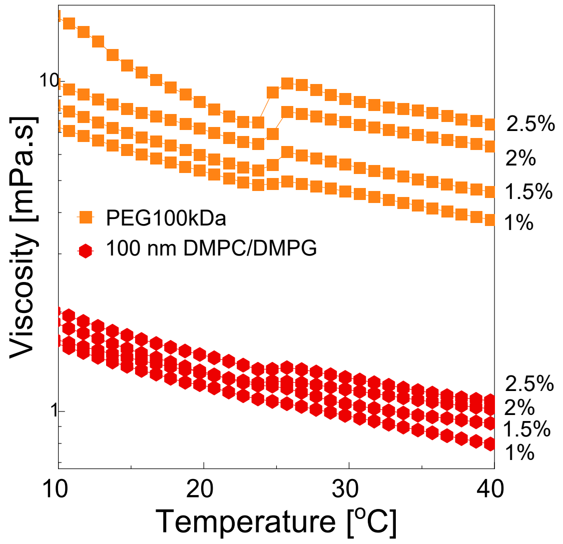

Figure S7. The mixture of 100 nm liposomes and PEG 100kDa solutions and 100 nm liposome solution at 2.5%, 2%, 1.5%, and 1% concentrations.

Volume Fraction Calculation

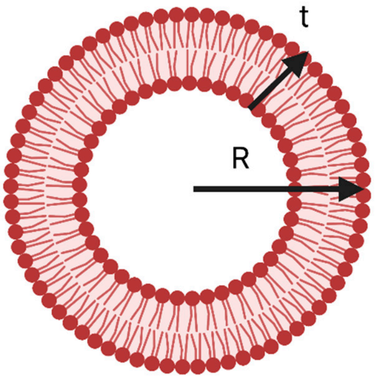

For 100 nm liposomes at 35°C and 2% concentration:

Hydrodynamic radius, R=$\frac{119.9nm}{2}\frac{119.9nm}{2}$= 59.95 nm,

Total surface area = $4\pi[R^2+(R-t)^2]$ where t is the bilayer thickness obtained from SAXS/WAXS.

Total number of lipid molecules per liposome,

N_{Total}= $\frac{4\pi[R^2+(R-t)^2]}{a}=\frac{4\pi[(59.59nm)^2+(59.95nm\ -6.28nm)^2]}{0.6nm^2}=135601$, where a is area per lipid. (a is equal to 0.6 nm^2 [2]for fluid phase, and 0.605nm^2 for gel phase[3].)

Total number of liposomes in solution:

$$N_{Liposomes}=\frac{M_{lipid}\times N_A}{N_{Total}}=\frac{0.027M\times6.02\times10^{23}/mol}{13560\times1000}=1.19\times10^{14}\,liposomes/mL$$

Volume of a single liposome:

$$V_{single\ Liposome}=\frac{4}{3}\pi R^3=\frac{4}{3}\pi(59.95)^3=902518.633nm^3$$

Total volume of liposomes:

$$V_{TotalLiposomes}=N_{Liposomes}\ \mathrm{X}\ V_{single\ Liposome}=1.08\ \mathrm{x}\ 10^{20}\ \mathrm{nm}^3$$

$$\text{Volume Fraction}=\frac{Vliposomes}{Vtotal}=\frac{1.39\times10^{20}\,nm^{3}}{1mL}\times\frac{1mL}{10^{21}\,nm^{3}}=0.108$$

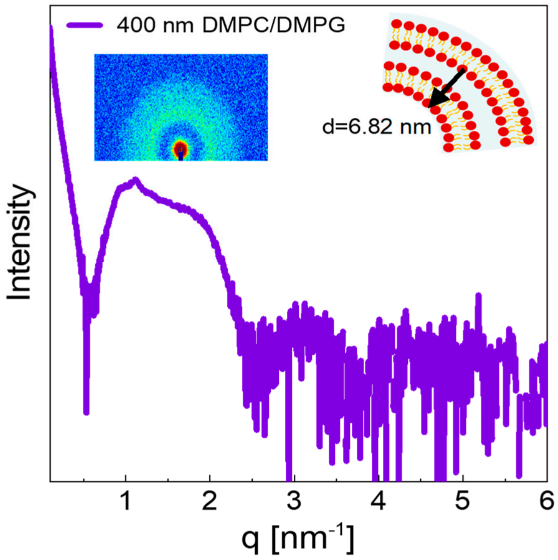

Figure S8. SAXS data of 400 nm DMPC/DMPG liposomes at 15ºC, inset is raw detector data. Bilayer thickness is obtained as 6.82 nm for the q*=0.92 nm^{-1}.

Table S3. Calculated Volume Fractions of 100-nm Liposomes and 100-nm Liposomes in PEG 100kDa solutions

	100nm Liposomes $\phi_{Liposomes}$		100nm Liposomes in PEG100kDa $\phi_{Liposomes}$	
Concentrations	35ºC	15ºC	35ºC	15ºC
0.08%	0.136	0.119	0.159	0.119
1.2%	0.108	0.095	0.136	0.103
1.7%	0.080	0.070	0.092	0.073
2.0%	0.052	0.046	0.064	0.048

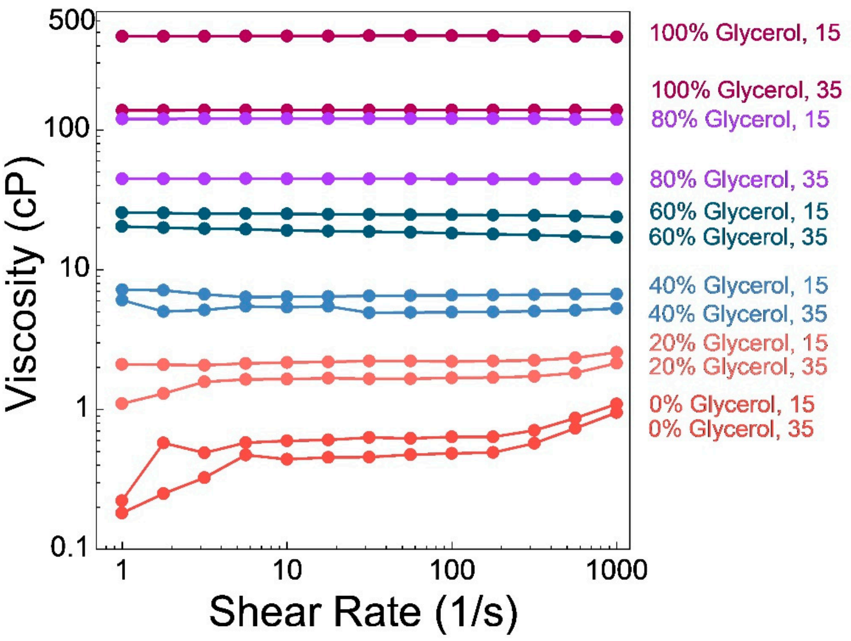

Figure S9. Viscosity of oil red o in glycerol and methanol mixtures as a function of shear rate at different glycerol compositions

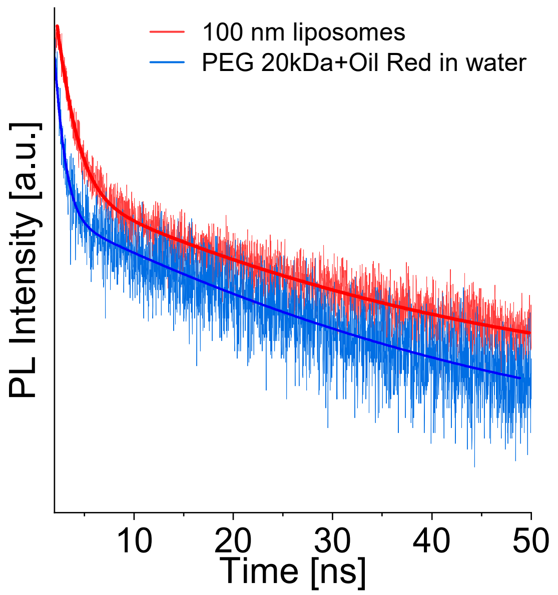

Figure S10. Time-resolved fluorescence decays of Oil-Red-O inside lipid bilayer, Oil-Red-O inside PEG20kDa and water solution at 35°C. Oil-Red-O concentration is kept constant for all measurements. The average fluorescence lifetime values for Oil-Red-O inside lipid bilayer, Oil-Red-O inside PEG20kDa and water solution at 35°C are measured as 28.9, and 12.7 ns, respectively.

Table S4. Fit parameters of time-resolved fluorescence lifetime of Oil-Red-O inside lipid bilayers in the absence and presence of PEG with various M_w's at 35 °C

	lifetime (ns) at 35°C					
	A_1	A_2	τ_1	τ_2	τ_{avg}	χ^2
100 nm liposomes	0.58	0.42	4.51±0.021	33.45±0.036	28.91±0.038	1.03
in PEG 1.5kDa	0.53	0.47	4.18±0.028	30.89±0.029	27.38±0.033	0.98
in PEG 20kDa	0.55	0.45	4.51±0.031	31.62±0.035	27.59±0.036	0.95
in PEG 100kDa	0.52	0.48	4.92±0.044	32.29±0.041	28.42±0.029	0.99
in PEG 400kDa	0.57	0.43	4.34±0.039	32.99±0.027	28.74±0.031	1.01

Table S5. Fit parameters of time-resolved fluorescence lifetime of Oil-Red-O inside lipid bilayers in the absence and presence of PEG with various M_w's at 15 °C

	lifetime (ns) at 15°C					
	A_1	A_2	τ_1	τ_2	τ_{avg}	χ^2
100 nm liposomes	0.54	0.46	3.85±0.012	27.42±0.048	24.01±0.032	0.97
in PEG 1.5kDa	0.52	0.48	3.73±0.019	23.50±0.039	20.61±0.013	1.01
in PEG 20kDa	0.53	0.48	3.51±0.015	24.34±0.045	21.43±0.022	0.99
in PEG 100kDa	0.51	0.49	4.17±0.023	25.83±0.028	22.72±0.019	0.96
in PEG 400kDa	0.52	0.48	3.82±0.020	26.98±0.034	23.91±0.028	0.98

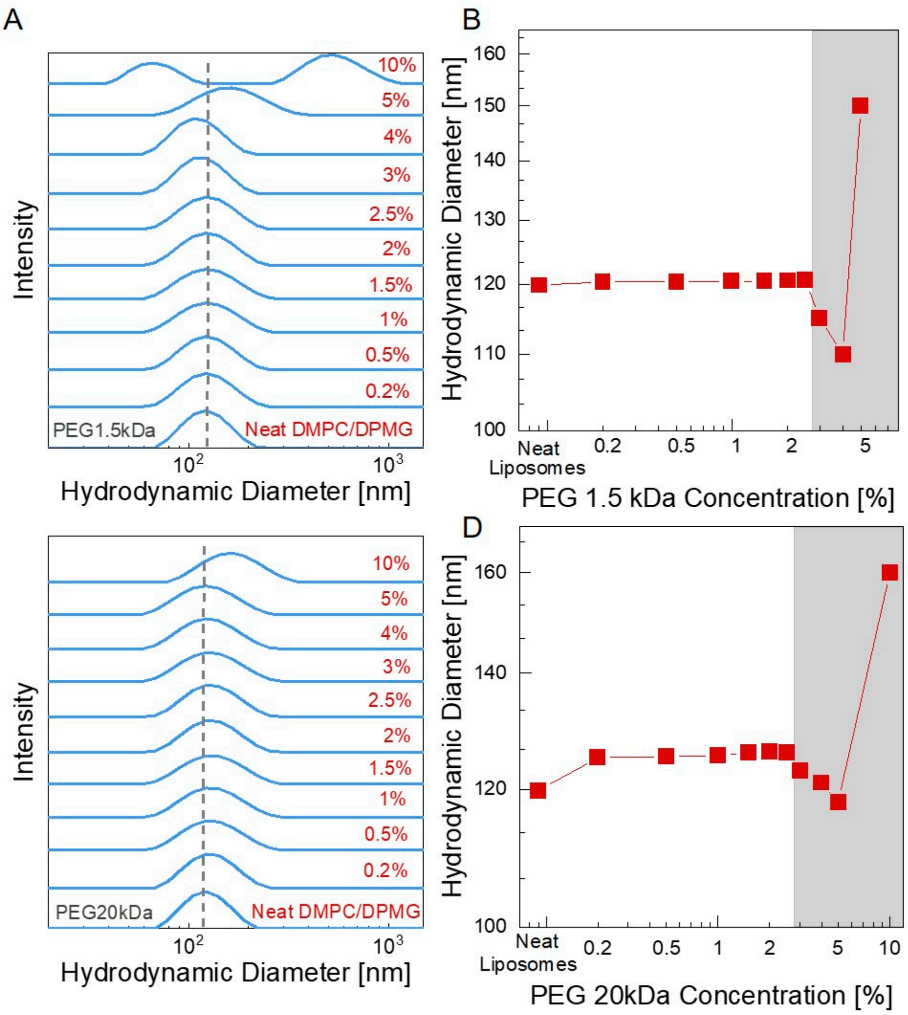

Figure S11. Hydrodynamic sizes liposomes in varying A. PEG 1.5kDa and B. PEG 20kDa concentrations from 0.2 to 10 w%. Gray-shaded area represents the concentration range in which the size of the liposomes starts decreasing.

Table S6. Zeta potential values of liposomes in varying PEG 1.5kDa and PEG 20kDa concentrations from 0.2 to 2.5 wt%.

PEG 1.5kDa		PEG 20kDa	
**PEG Concentration (%)**	**Zeta Potential (mV)**	**PEG Concentration (%)**	**Zeta Potential (mV)**
**0**	-9.78±1.32	**0**	-9.78±1.32
**0.2**	-9.11±1.01	**0.2**	-4.34±0.91
**0.5**	-2.53±0.53	**0.5**	-2.51±0.47
**1**	-1.05±0.23	**1**	-1.48±0.27
**1.5**	-0.952±0.07	**1.5**	-1.18±0.12
**2**	-0.360±0.04	**2**	-0.204±0.02
**2.5**	-0.150±0.01	**2.5**	-0.209±0.01

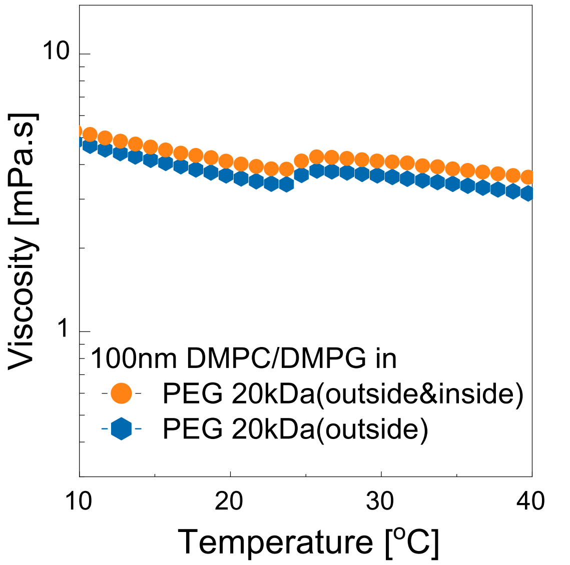

Figure S12. The comparison of the viscosity of 100 nm liposomes in PEG 20 kDa solution where PEG chains are outside and both outside and inside (elimination of osmotic pressure)

References

(1) Fetters, L. J.; Lohse, D. J.; Richter, D.; Witten, T. A.; Zirkel, A. Connection between Polymer Molecular Weight, Density, Chain Dimensions, and Melt Viscoelastic Properties. *Macromolecules* **1994**, *27* (17), 4639-4647. DOI: 10.1021/ma00095a001.

(2) Kučerka, N.; Nieh, M.-P.; Katsaras, J. Fluid phase lipid areas and bilayer thicknesses of commonly used phosphatidylcholines as a function of temperature. *Biochimica et Biophysica Acta (BBA) - Biomembranes* **2011**, *1808* (11), 2761-2771. DOI: https://doi.org/10.1016/j.bbamem.2011.07.022.

(3) Drabik, D.; Chodaczek, G.; Kraszewski, S.; Langner, M. Mechanical Properties Determination of DMPC, DPPC, DSPC, and HSPC Solid-Ordered Bilayers. *Langmuir* **2020**, *36* (14), 3826-3835. DOI: 10.1021/acs.langmuir.0c00475.
